# Supplementary material for: Mechanistic insights into the regulation of plant phosphate homeostasis by the rice SPX2 – PHR2 complex
Source: Nat Commun. 2022 Mar 24;13:1581. doi: 10.1038/s41467-022-29275-8 (PMC8948245; doi:10.1038/s41467-022-29275-8)
Supplement: Supplementary file 1 — Supplementary Information [file 41467_2022_29275_MOESM1_ESM.pdf]

Supplementary Information

Mechanistic insights into the regulation of plant phosphate homeostasis by the rice SPX2 – PHR2 complex

Zeyuan Guan, Qunxia Zhang, Zhifei Zhang *et al*.

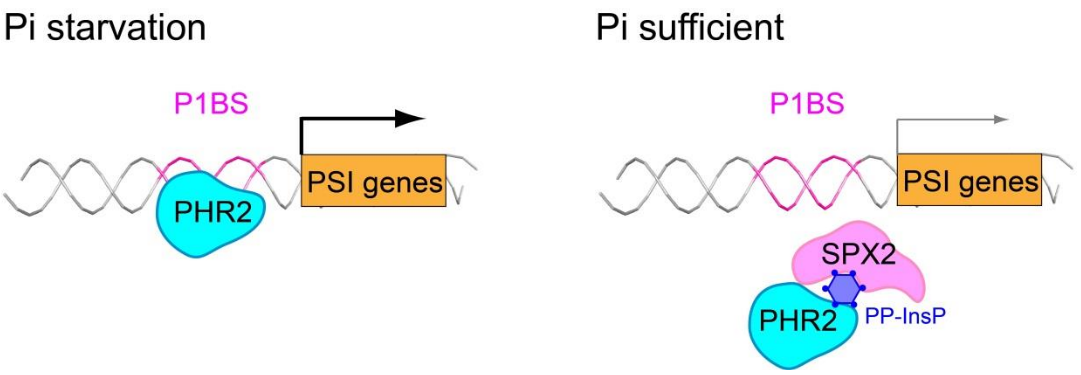

Supplementary Fig. 1. Regulation of PSI genes expression under Pi starvation and sufficient Pi conditions, respectively. PP-InsP is proposed to be a "molecular glue" mediating the association of PP-InsP receptor and interacting protein[1].

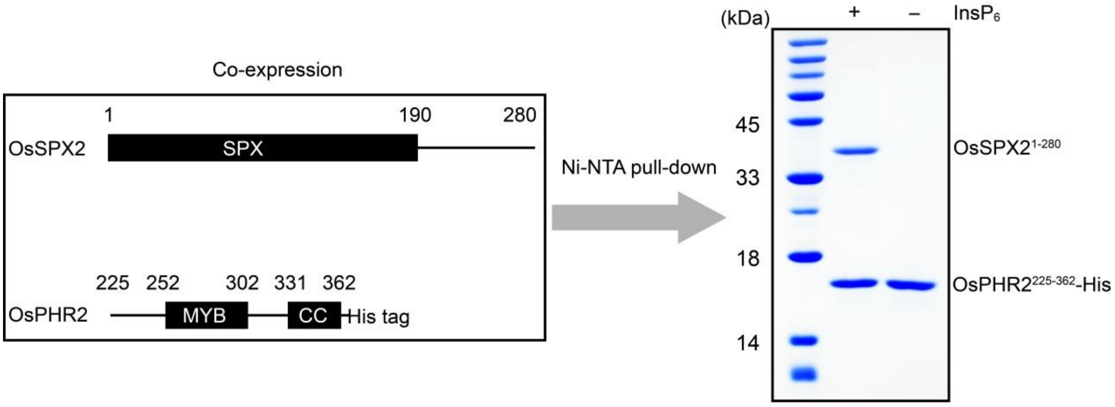

Supplementary Fig. 2. The association of rice SPX2 and rice PHR2 is $InsP_6$ dependent. Domain organizations of SPX, MYB and CC in rice SPX2 and PHR2 are schematically illustrated in the left frame. In the presence of $InsP_6$, the SPX2 can be co-eluted from Ni-NTA beads with His-tagged PHR2 (right frame). In contrast, SPX2 cannot be pulled down by His-tagged PHR2 in the absence of $InsP_6$. These results indicated that the interaction between SPX2 and PHR2 is $InsP_6$ dependent. The positions of SPX2 and PHR2 in the SDS-PAGE gel are highlighted, respectively. Experiments were independently repeated three times with similar results.

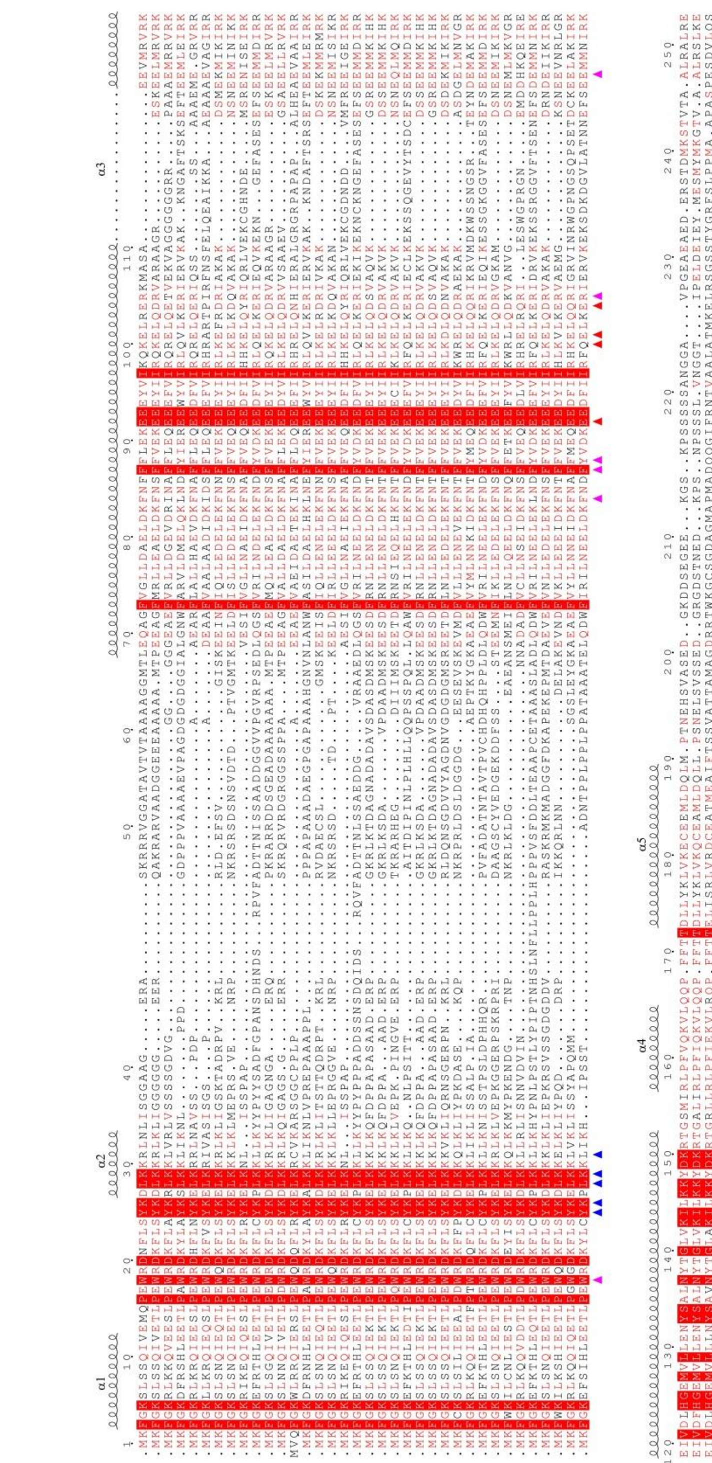
InsP6 binding
OsSPX2 dimerization
OSPHR2 binding

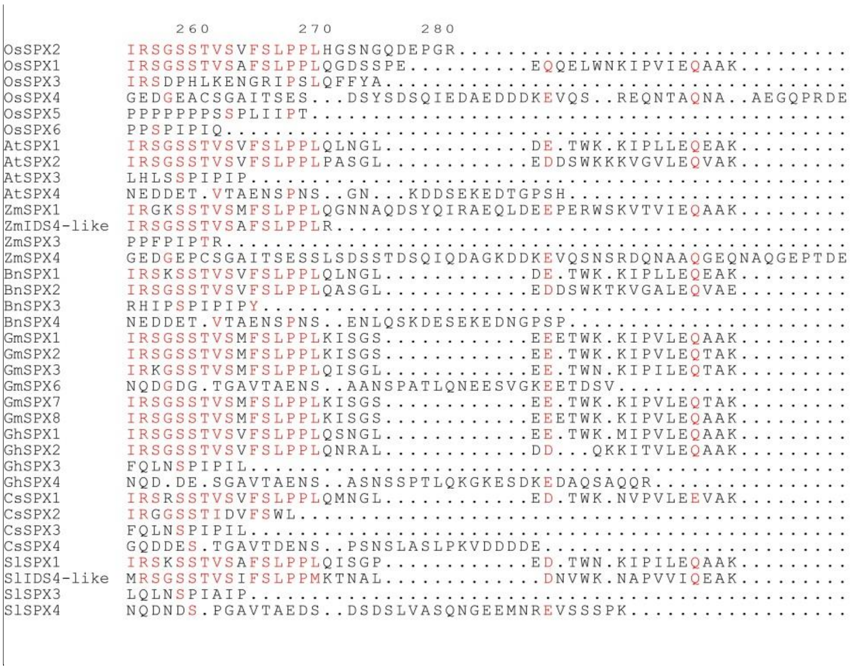

Supplementary Fig. 3. Sequence alignment of stand-alone SPX proteins. The sequence of *Oryza Sativa* SPX2 is aligned with other homologs. The alignment is generated using the MultAlin[2]. The sequence identity is indicated by white letters against a red background, and the sequence of a similarity over 90% is indicated by red letters. The secondary elements of rice SPX2 are labeled at the top of the alignment. The residues responsible for $InsP_6$ binding, rice SPX2 dimerization and rice PHR2 binding are indicated with blue, magenta and red triangles at the bottom of the alignment, respectively.

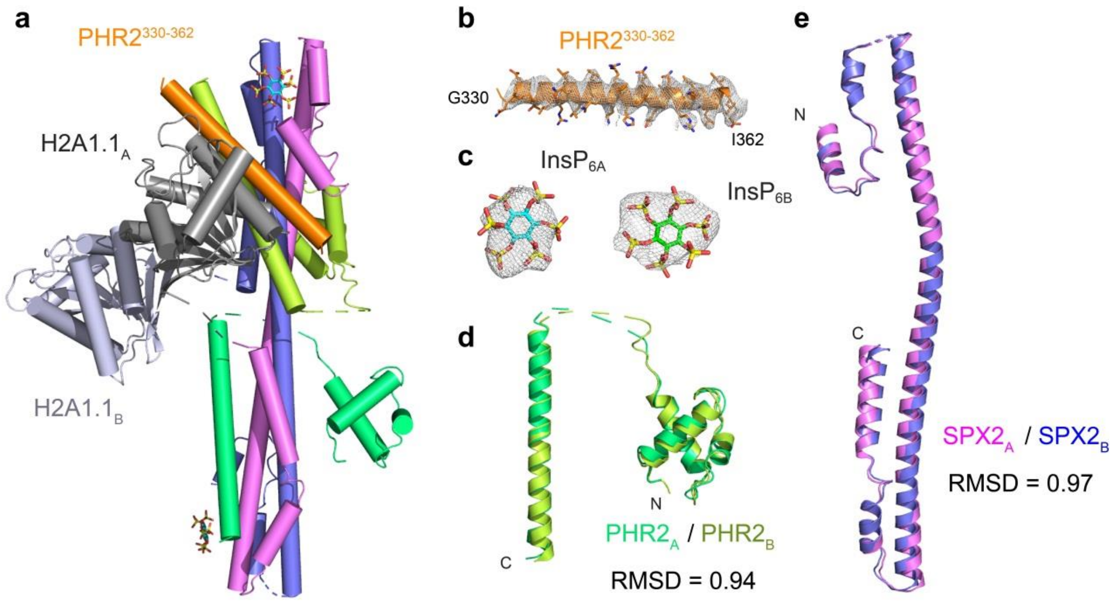

Supplementary Fig. 4. Overall structure of SPX2$^{1\text{-}202/\Delta47\text{-}59}$/InsP$_6$/PHR2$^{225\text{-}362}$ complex and particular elements presentation. a, The overall structure of mH2A1.1 fused SPX2$^{1\text{-}202/\Delta47\text{-}59}$ in complex with InsP$_6$ and PHR2$^{225\text{-}362}$. The SPX2$^{1\text{-}202//\Delta47\text{-}59}$, InsP$_6$, and PHR2$^{225\text{-}362}$ are colored in the same scheme as Fig. 1a in the main text. The mH2A1.1 tags are colored in gray cartoon representation. In the crystal, some extra densities can be unambiguously assigned to a PHR2 fragment that comprises the residues 330-362 of the CC domain. This PHR2$^{330\text{-}362}$ fragment is colored in orange cartoon representation. **b**, The composite omit map fits well with the PHR2$^{330\text{-}362}$ fragment. The 2Fo-Fc omit electron density map is contoured at 1.0 σ. The extra CC domain in the crystal may be degraded from the PHR2$^{225\text{-}362}$ (involves the MYB domain and CC domain) during crystallization process. Previous results have also indicated that the CC domain is easily cleaved from PHR2 due to proteolysis during crystallization process[3,4]. For clearer visualization, the polypeptides of mH2A1.1 tag and PHR2$^{330\text{-}362}$ fragment are omitted in the main text presentation. **c**, The 2Fo-Fc omit electron density map of the two InsP$_6$ molecules. It is contoured at 1.5 σ. **d**, The structures of the two PHR2 protomers and, **e**, the two SPX2 protomers in the ternary complex are almost identically with a RMSD of 0.94 and 0.97, respectively.

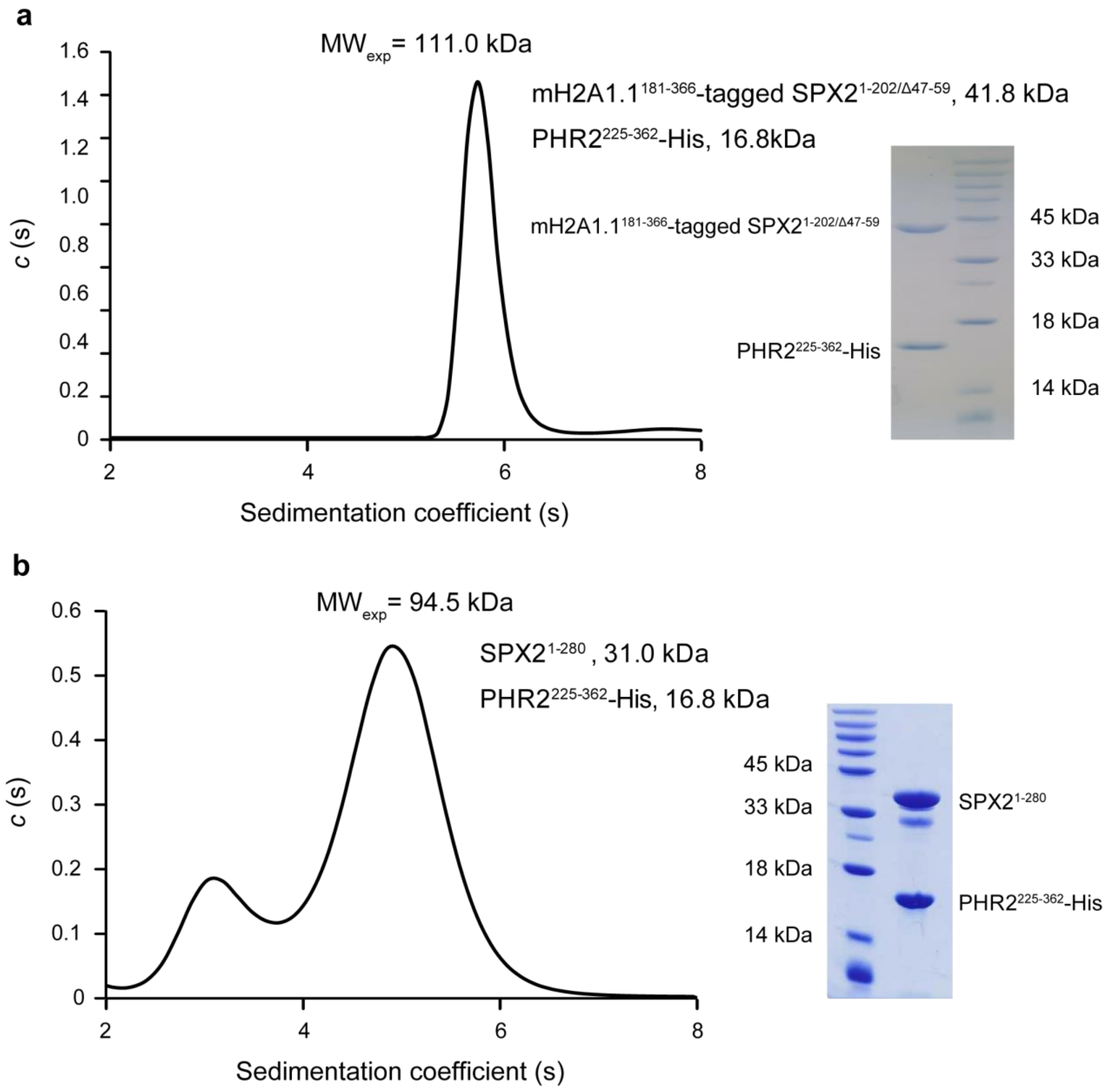

Supplementary Fig. 5. Analytical ultracentrifugation (AUC) characterizations. **a**, The measured molecular weight of the crystallized complex (mH2A1.1$^{181-366}$-tagged SPX2$^{1-202/\Delta47-59}$/InsP$_6$/PHR2$^{225-362}$ complex) in solution is 111.0 kDa, that is about twice of the sum of mH2A1.1$^{181-366}$-tagged SPX2$^{1-202/\Delta47-59}$ and PHR2$^{225-362}$ (58.6 kDa in sum). It indicates that the SPX2 binds to PHR2 with a stoichiometry ratio of 2:2, and it is consisted with the crystal structure. **b**, For the molecular measurement of the full-length SPX2$^{1-280}$ in the complex of PHR2$^{225-362}$, the major peak of experimentally measured molecular weight is 94.5 kDa, that is also about twice of the sum of the full-length SPX2$^{1-280}$ and PHR2$^{225-362}$ (47.8 kDa in sum). This indicates that the mH2A1.1$^{181-366}$ fusion tag and the loop truncations have little impact on the SPX2 structure and its ability to bind PHR2. The experiment was performed using the purified complex in the presence of 1 mM InsP$_6$. The smaller species in the AUC result of **b** may be contributed from some proteolysis or contamination. Samples used for AUC experiments were visualized in the SDS-PAGE gels, respectively. All the experiments were independently repeated three times with similar results.

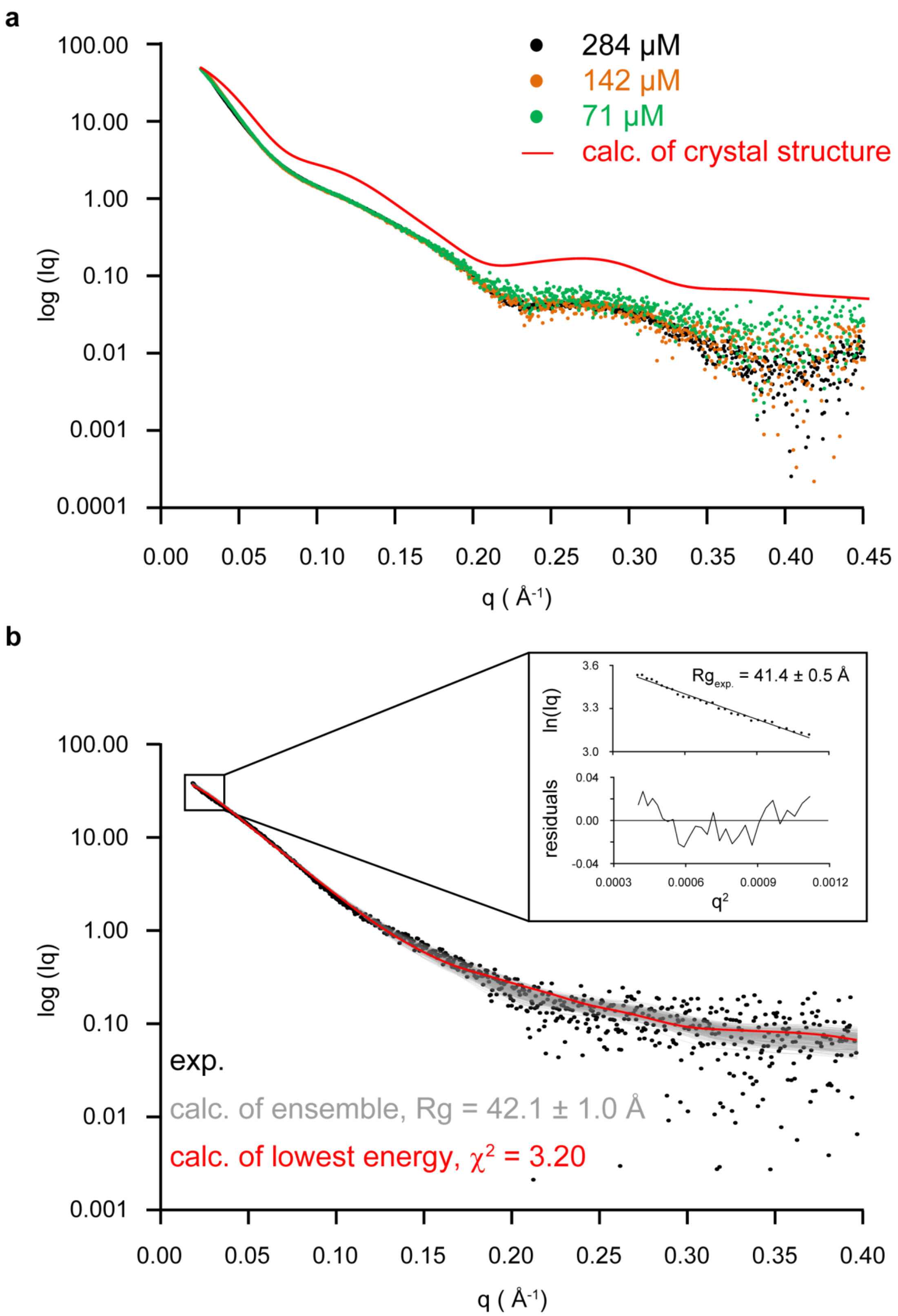

Supplementary Fig. 6. Small-angle X-ray scattering (SAXS) characterizations. a, SAXS characterizes the structure of crystallized mH2A1.1$^{181\text{-}366}$-tagged SPX2$^{1\text{-}202/\Delta 47\text{-}59}$/InsP$_6$/PHR2$^{225\text{-}362}$ complex in solution. SAXS measurement was performed at 284 µM (black dots), 142 µM (orange dots), 71 µM (green dots), respectively. The three experimental scattering data were scaled by the first point. The theoretical scattering profile of the crystal structure (red line) was calculated using

CRYSOL and scaled by the first point of the experimental scattering data. SAXS data were collected in the presence of 1 mM $InsP_6$. **b**, SAXS characterizes the structure of $SXP2^{1-202}/InsP_6/PHR2^{225-362}$ complex in solution. The invisible residues and the deleted internal-residues in the crystal structure were patched and optimized for the calculation of structure-derived theoretical SAXS profile (See Methods). 260 conformations were optimized to account the flexibility of theses residues, and the theoretical SAXS profiles of this conformation ensemble (gray line) were calculated and plotted against the experimental data (black dots). It shows that there is no significant difference between the calculated profiles and experimental data. Furthermore, one optimized conformation (red line), with the lowest energy, fits well with the experimental data of $SPX2^{1-202}/InsP_6/PHR2^{225-362}$ complex in solution (black dots). The chi square (χ^2) for the fitting is 3.20. Guinier plot using experimental small-angle scattering data shows that the radius of gyration (Rg) is 41.4 ± 0.5 Å (insert). It is in line with the theoretical calculated Rg of optimized conformation ensemble (42.1 ± 1.0 Å). Taken together, it indicates that the crystal structure is maintained in solution, and that the internal-residues deletion (Δ47-59) and $mH2A1.1^{181-366}$ fusion of SPX2 have little impact on its structure and PHR2 association. The experiment was performed for the purified $SPX2^{1-202}/PHR2^{225-362}$ complex (no mH2A1.1 tag and no internal-residues deletion) in the presence of 1 mM $InsP_6$.

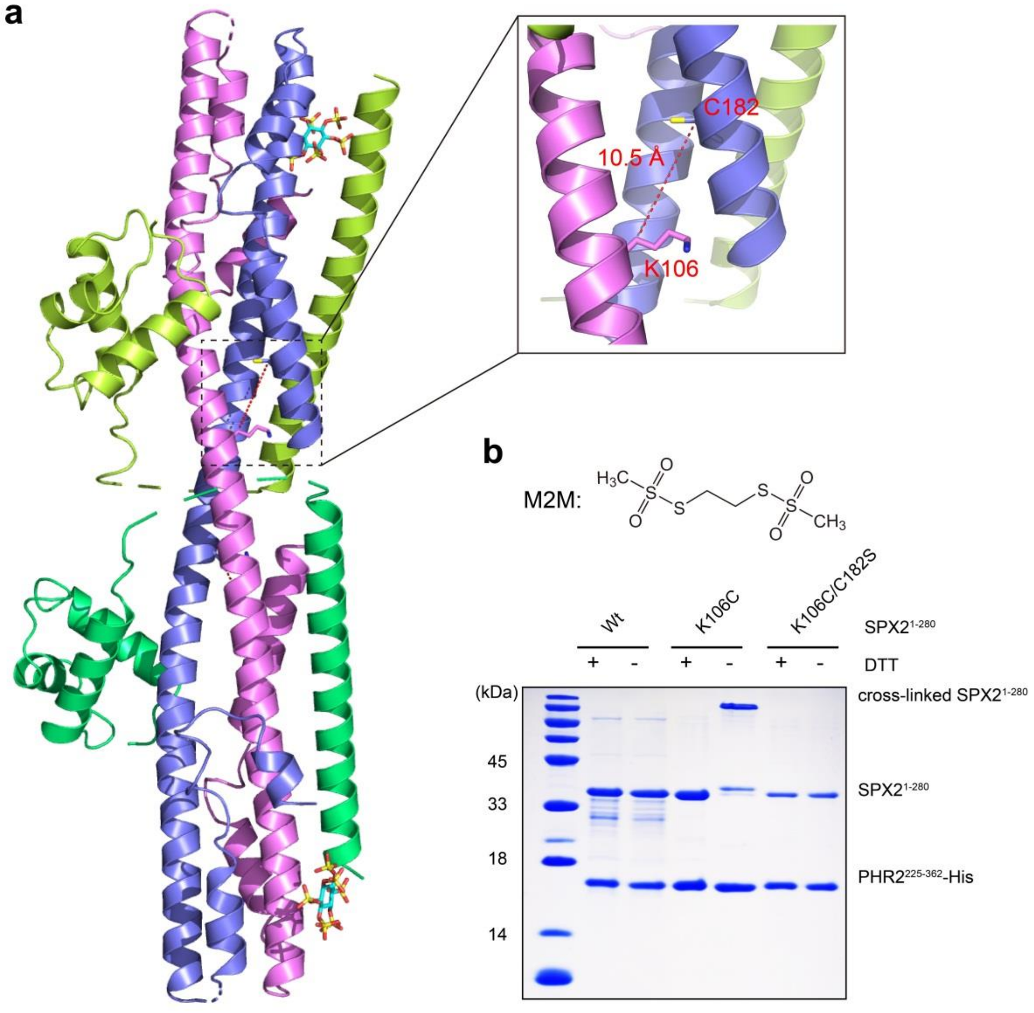

Supplementary Fig. 7. Thiol-directed chemical crosslinking validates the domain-swapped dimeric conformation of SPX2 in the SPX2/$InsP_6$/PHR2 complex. a, Representation of two

spatially adjacent residues, K106 and C182, in the two protomers of the domain-swapped SPX2 dimer. The Cβ-Cβ distance between K106 and C182 is 10.5 Å. **b**, SDS-PAGE imaging of thiol-directed chemical crosslinking. The M2M (1,2-ethanediyl bismethanethiosulfonate) probe can crosslink two cysteine residues within a Cβ-Cβ distance of 11 Å. By mutating K106 of SPX2 into a cysteine, two distinct SPX2 molecules in the SPX2/InsP$_6$/PHR2 complex were cross-linked by M2M, and the cross-linked SPX2 can be reversibly reduced by DTT (dithiothreitol). Neither the wild-type SPX2, possesses the endogenous C182, nor the K106C/C182S double mutant can be cross-linked. Thus, the thiol-directed chemical crosslinking results corroborate the domain-swapped dimeric conformation of SPX2 observed in the SPX2/InsP$_6$/PHR2 complex structure. The crosslinking experiments were performed using the purified SPX2$^{1\text{-}280}$/PHR2$^{225\text{-}362}$ complex with particular mutations in the presence of 1 mM InsP$_6$. Experiments were independently repeated three times with similar results.

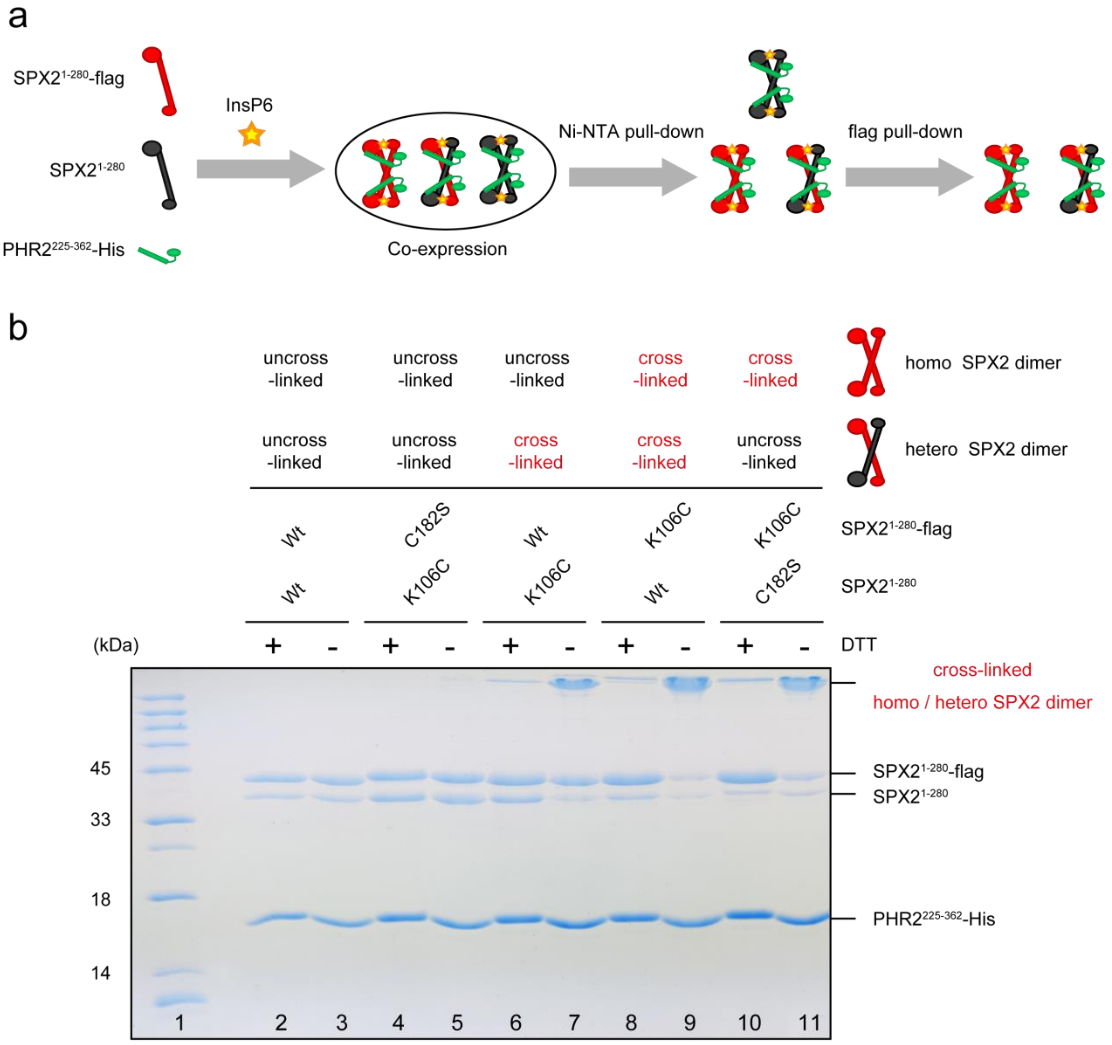

Supplementary Fig. 8. Encoding K106C and C182S mutation on two distinct SPX2 molecules for thiol-directed chemical crosslinking to validate the domain-swapped SPX2 dimer in the SPX2/InsP$_6$/PHR2 complex. a, Diagram illustrates the preparation of PHR2 in complex with SPX2-flag/SPX2-flag homodimer and SPX2-flag/SPX2 heterodimer for crosslinking experiments. **b,** SDS-PAGE imaging of thiol-directed chemical crosslinking. The mixture of PHR2 in complex with SPX2-flag/SPX2-flag homodimer and SPX2-flag/SPX2

heterodimer with / without specific mutations were subjected for M2M crosslinking. Experiments were independently repeated three times with similar results.

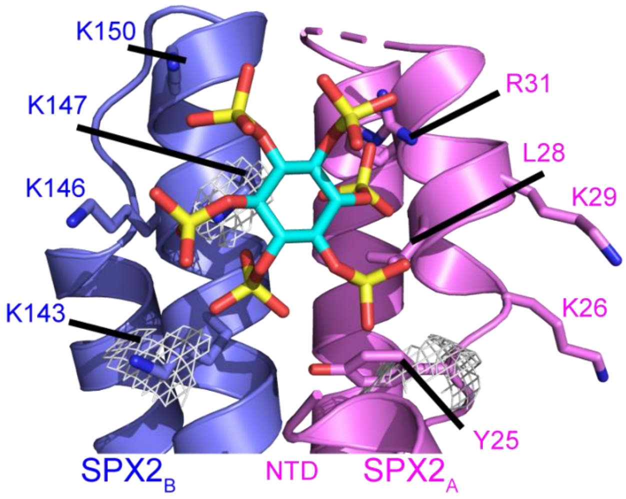

Supplementary Fig. 9. The 2Fo-Fc omit electron density map of critical residues for $InsP_6$ recognition. It is contoured at 1.0 σ. The densities of these residues are poor, that may be due to the low resolution of the structure.

a

OsSPX2	E93	E104	K100	E101	H124	E131	E183	D187
OsSPX4	E87	E98	Q94	V95	H132	Q139	E191	E195

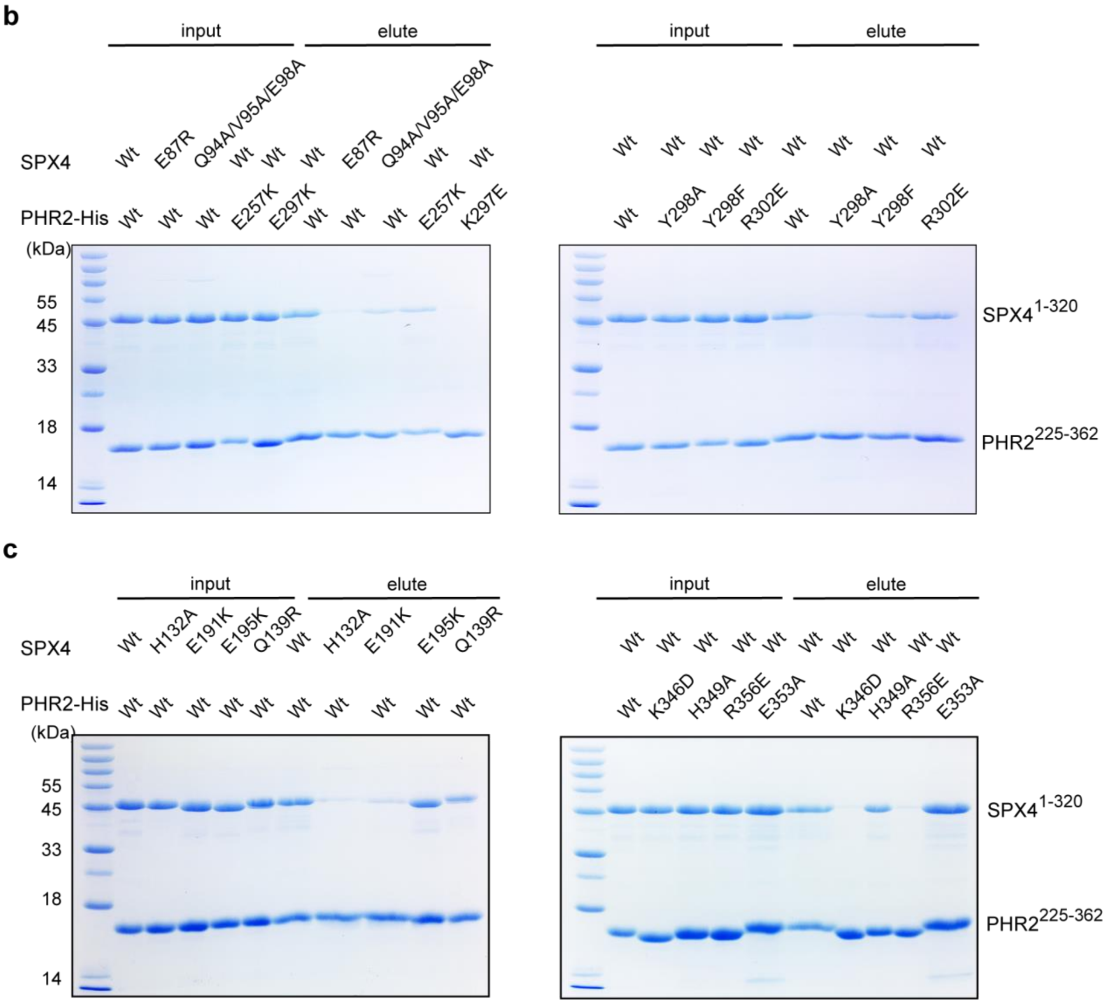

Supplementary Fig. 10. Pull-down analysis assesses the conserved interface residues contributing for the interaction between rice SPX4 and PHR2. a, Conserved key residues in rice SPX2 and SPX4 required for PHR2 binding. *In vitro* Ni-NTA pull-down characterizes the perturbed interactions between full-length SPX4$^{1\text{-}320}$ and His-tagged PHR2$^{225\text{-}362}$, upon residues mutation in the interface between SPX4 and, **b**, PHR2 ^{MYB} domain and, **c**, PHR2 ^{CC} domain, respectively. Different mutated versions of the full-length SPX4$^{1\text{-}320}$ and His-tagged PHR2$^{225\text{-}362}$ was prepared separately for the pull-down assay. All the experiments were independently repeated three times with similar results.

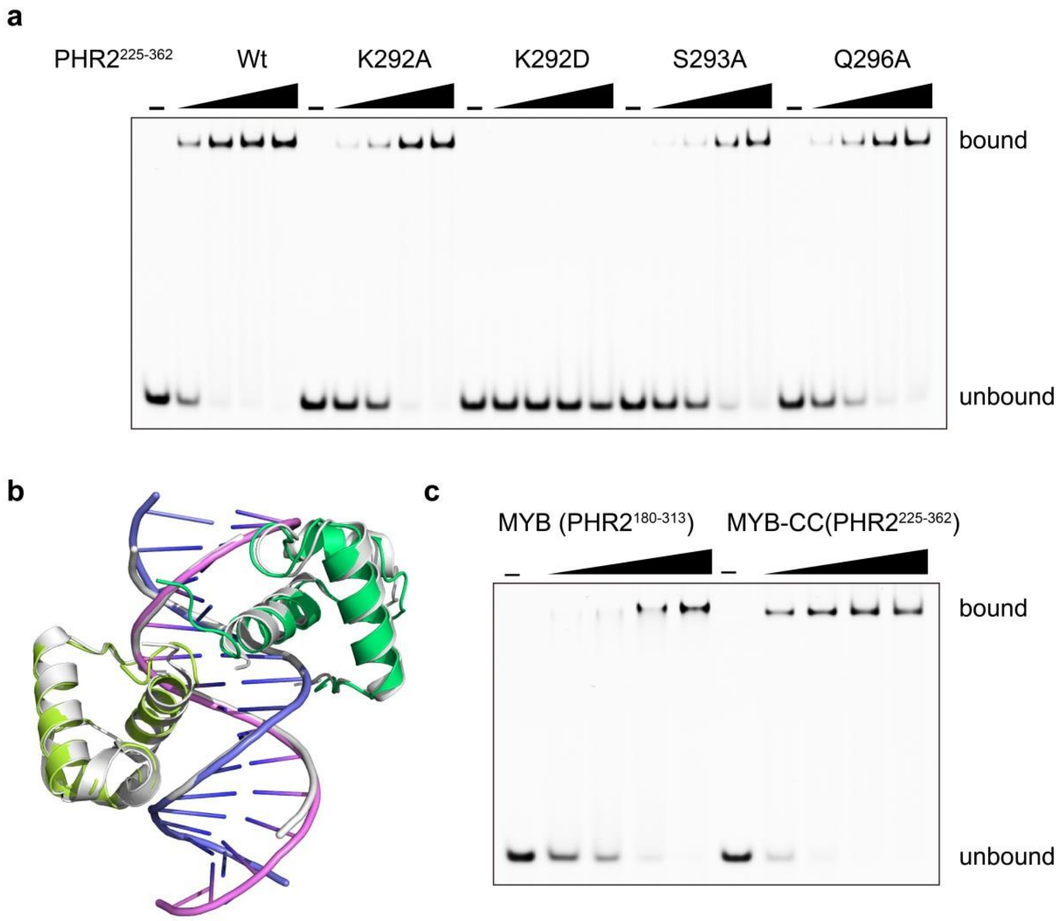

Supplementary Fig. 11. Characterize the recognition of P1BS motif by rice PHR2. a, Electrophoretic mobility shift assay (EMSA) assessed the key residues of rice PHR2 for the recognition of P1BS. The boundary of PHR2$^{225\text{-}362}$ comprising the MYB and CC domains was used for the EMSA experiments. **b**, Structural comparison between the rice MYB^{PHR2}/P1BS complex and *Arabidopsis* MYB^{PHR1}/P1BS complex. Rice MYB^{PHR2}/P1BS structure is colored in the same scheme as Fig. 4a in the main text, and *Arabidopsis* MYB^{PHR1}/P1BS structure is colored in gray cartoon representation. **c**, EMSA results showed that the rice PHR2$^{225\text{-}362}$ comprising both MYB and CC domains has stronger binding affinity to P1BS than the only MYB domain (PHR2$^{180\text{-}313}$). All the experiments were independently repeated three times with similar results.

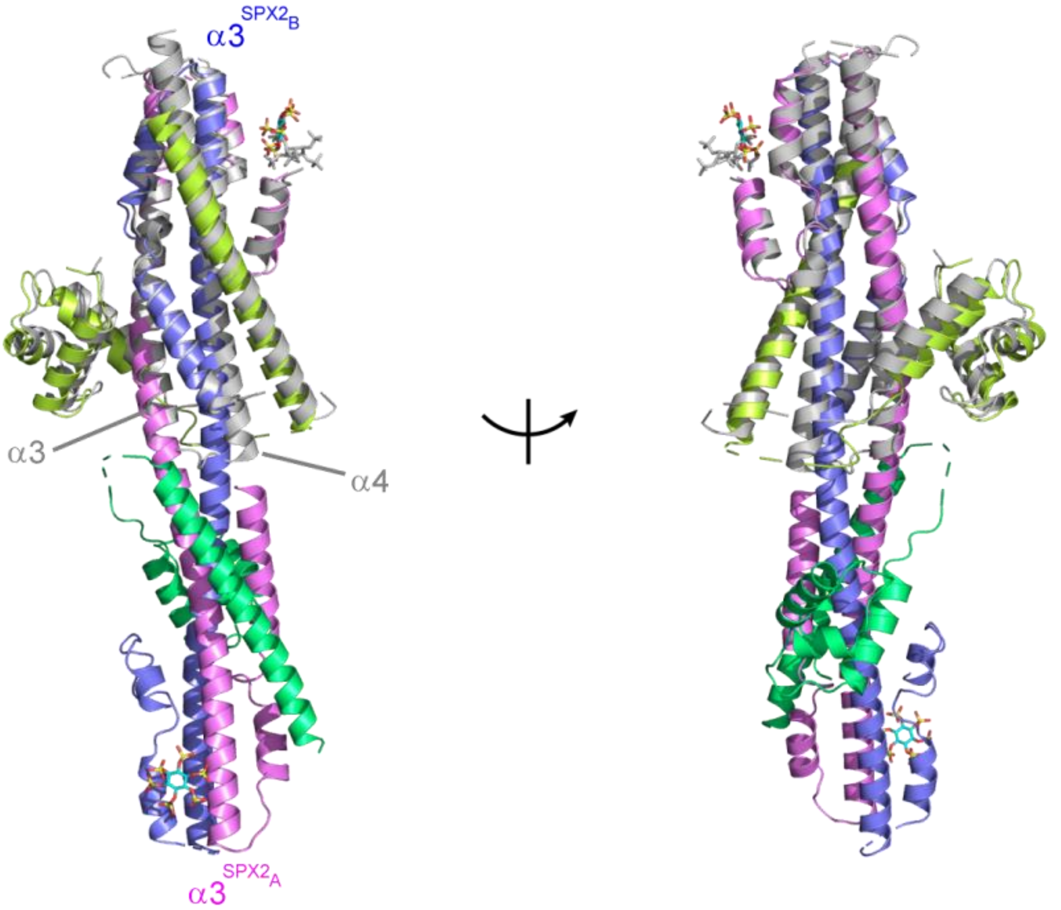

Supplementary Fig. 12. Structural comparison between the rice SPX2$^{1\text{-}202/\Delta47\text{-}59}$/InsP$_6$/PHR2$^{225\text{-}362}$ complex and SPX1$^{1\text{-}198}$/InsP$_6$/PHR2$^{248\text{-}380}$ complex. SPX2$^{1\text{-}202/\Delta47\text{-}59}$/InsP$_6$/PHR2$^{225\text{-}362}$ structure is colored in the same scheme as Fig. 1a in the main text, and SPX1$^{1\text{-}198}$/InsP$_6$/PHR2$^{248\text{-}380}$ structure (7E40.PDB) is colored in gray. Core helices α3 and α4 of SPX1, and two antiparallel extended helices α3 of domain-swapped SPX2 are indicated.

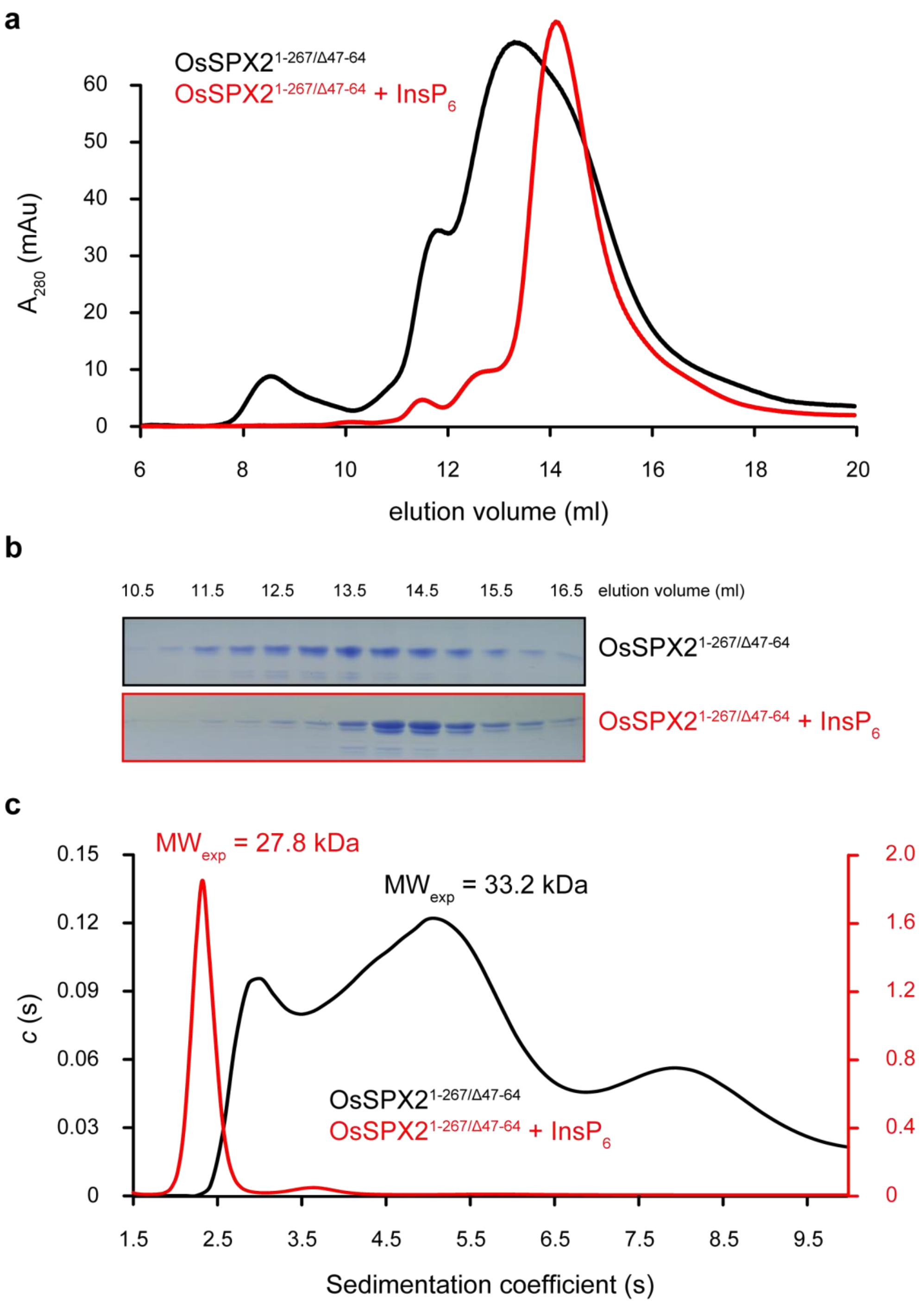

Supplementary Fig. 13. Clarifying if the $InsP_6$ binding can induce SPX2 dimerization by SEC and AUC. a, SEC profiles of $SPX2^{1-267/\Delta 47-64}$ in the absence and in the presence of 1 mM $InsP_6$ are colored in black and red, respectively. Peak fractions were visualized using Coomassie-blue stained SDS-PAGE in **b**. SEC assay was performed using Superdex-200 Increase 10/300 column. Experiments were independently repeated three times with similar results. **c**, The AUC measured molecular weight of $SPX2^{1-267/\Delta 47-64}$ is 33.2 kDa and 27.8 kDa in the absence and in the presence of 1 mM $InsP_6$, respectively.

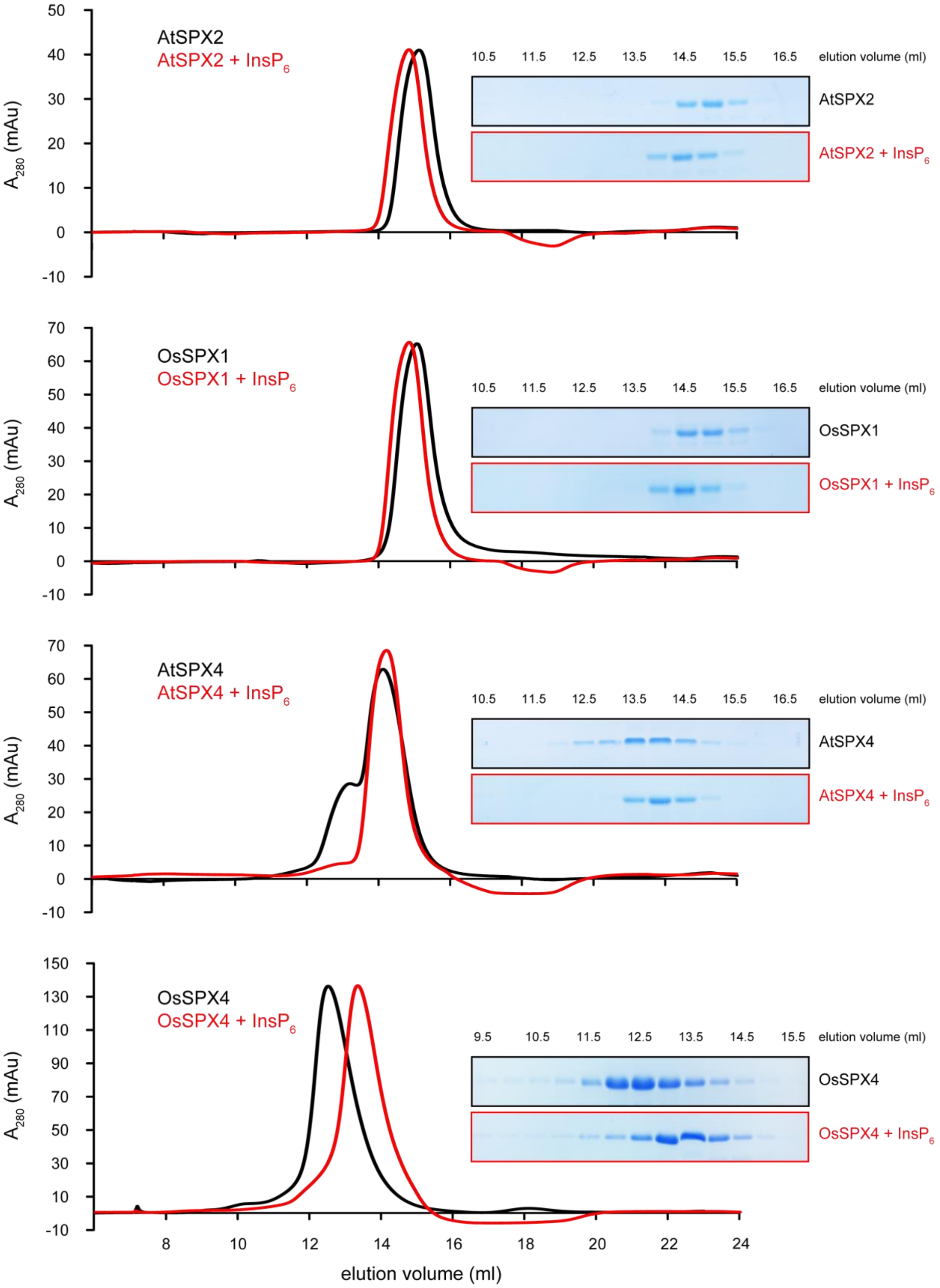

Supplementary Fig. 14. Assessing $InsP_6$-induced conformational changes of other plant stand-alone SPX proteins. SEC profiles of SPX proteins in the absence and in the presence of 1 mM $InsP_6$ are colored in black and red, respectively. Peak fractions were visualized using Coomassie-blue stained SDS-PAGE (insert). After adding InsP6, the elution volume of AtSPX2 and OsSPX1 moved forward, AtSPX4 changed little and OsSPX4 shifted later, respectively. SEC was performed using Superdex-200 Increase 10/300 column. All the experiments were independently repeated three times with similar results.

Supplementary Table 1. Statistics of crystal data collection and structures refinement.

structure	SPX2$^{1-202/\Delta47-59}$/InsP6/PHR2$^{225-362}$ (PDB CODE: 7D3Y)	PHR2$^{180-313}$/DNA (PDN CODE: 7D3T)
**Data collection**		
Space Group	P3$_1$ 2 1	P 1 2$_1$ 1
Unit cell a, b, c,	147.81, 147.81, 143.29	57.41, 100.82, 79.35
α, β, γ (Å) (°)	90.00, 90.00, 120.00	90.00, 102.46, 90.00
Wavelength (Å)	0.9792	0.9785
Resolution (Å)	45~3.11 (3.28~3.11)	49~2.70 (2.83~2.70)
R_{merge} (%)	4.9 (84.4)	6.3 (103.8)
R_{pim} (%)	1.2 (18.8)	4.5 (69.1)
CC(1/2) (%)	99.9 (95.7)	99.7 (98.1)
I/σ	35.4 (4.7)	12.0 (1.7)
Completeness (%)	99.9 (95.7)	99.7 (98.1)
Number of measured reflections	653,746 (99,405)	81,972 (11,093)
Number of unique reflections	32,993 (4,743)	24,283 (3,197)
Redundancy	19.8 (21.0)	3.4 (3.5)
Wilson B factor (Å^2)	110.1	44.5
**Refinement**		
R_{work} /R_{free} (%)	22.02/25.26	21.28/25.78
**Number of atoms**		
Protein main chain	3640	956
Protein side chain	3681	1015
Protein all atoms	7321	1971
Water molecules	0	9
Other entities	72	1309
All atoms	7393	3289
**Average B value (Å^2)**		
Protein main chain	141.6	78.3
Protein side chain	157	84.0
Protein all atoms	146.2	84.3
Water molecules		68.5
Other entities	280.2	77.2
All atoms	147.5	79.6
**Rms deviations from ideal values**		
Bonds (Å)	0.007	0.011
Angle (°)	0.563	1.376
**Ramachandran plot statistics (%)**		
Most favorable	98.09	94.8
Additionally allowed	1.92	5.2
Generously allowed	0	0
Disallowed	0	0
**MolProbity score**	1.03	1.91

Values in parentheses are for the highest resolution shell. $R_{merge}=\Sigma_h\Sigma_i|I_{h,i}-I_h|/\Sigma_h\Sigma_i I_{h,i}$, where I_h is the mean intensity of the *i* observations of symmetry related reflections of *h*. $R=\Sigma|F_{obs}-F_{calc}|/\Sigma F_{obs}$, where F_{calc} is the calculated protein structure factor from the atomic model (R_{free} was calculated with 5% of the reflections selected).

Supplementary Table 2. Primers used in this study.

Primers	Primer sequences (5′–3′)
**PCR products were cloned into pET21B vector**	
PHR2$_{225\text{-}362}$-Fw	AAGAAGGAGATATACATATGAGCGGTGAACCGAGCGCA GTTGCC
PHR2$_{225\text{-}362}$-Rv	TGGTGATGGTGATGCTCGAGAATGCGCAGCTGCAGTGA ACGCTG
PHR2$_{180\text{-}313}$-Fw	AAGAAGGAGATATACATATGAATGATTTTATGAATTATGA TTG
PHR2$_{180\text{-}313}$-Rv	TGGTGATGGTGATGCTCGAGTTCGCTGCTGCCTTCGCTC AGTTC
PHR2_ R250A-Fw	GTAATTCTAAAACCCGCATGGCGTGGACCCCGGAACTG CATG
PHR2_ R250A-Rv	CATGCAGTTCCGGGGTCCACGCCATGCGGGTTTTAGAAT TAC
PHR2_ E257K-Fw	GACCCCGGAACTGCATAAACGTTTTGTTGATGC
PHR2_ E257K-Rv	GCATCAACAAAACGTTTATGCAGTTCCGGGGTC
PHR2_ H294A-Fw	CCATTTATCATGTGAAAAGTGCGCTGCAGAAATATCGCA C
PHR2_ H294A-Rv	GTGCGATATTTCTGCAGCGCACTTTTCACATGATAAATGG
PHR2_ K297E-Fw	GAAAAGTCATCTGCAGGAATATCGCACCGCCCG
PHR2_ K297E-Rv	CGGGCGGTGCGATATTCCTGCAGATGACTTTTC
PHR2_ H294A/K297A-Fw	CCATTTATCATGTGAAAAGTGCGCTGCAGGCGTATCGCA CCGCCCGTTATC
PHR2_ H294A/K297A-Rv	GGTAAATAGTACACTTTTCACGCGACGTCCGCATAGCGT GGCGGGCAATAG
PHR2_ Y298A-Fw	GAAAAGTCATCTGCAGAAAGCGCGCACCGCCCGTTATC GTC
PHR2_ Y298A-Rv	GACGATAACGGGCGGTGCGCGCTTTCTGCAGATGACTTT TC
PHR2_ Y298F-Fw	GAAAAGTCATCTGCAGAAATTTCGCACCGCCCGTTATCG TC
PHR2_ Y298F-Rv	GACGATAACGGGCGGTGCGAAATTTCTGCAGATGACTTT TC
PHR2_ R302E-Fw	CAGAAATTTCGCACCGCCGAATATCGTCCGGAACTGAG C
PHR2_ R302E-Rv	GCTCAGTTCCGGACGATATTCGGCGGTGCGAAATTTCTG
PHR2_ K346D-Fw	CGCCTGCAGCTGGAACTGGATAAACGCCTGCATGAACA G
PHR2_ K346D-Rv	CTGTTCATGCAGGCGTTTATCCAGTTCCAGCTGCAGGCG
PHR2_ H349A-Fw	GAACTGCAGAAACGCCTGGCGGAACAGCTGGAAATTCA G
PHR2_ H349A-Rv	CTGAATTTCCAGCTGTTCCGCCAGGCGTTTCTGCAGTTC

PHR2_ R356E-Fw	GAACAGCTGGAAATTCAGGAATCACTGCAGCTGCGCATTG
PHR2_ R356E-Rv	CAATGCGCAGCTGCAGTGATTCCTGAATTTCCAGCTGTTC
PHR2_ E353A-Fw	CGCCTGCATGAACAGCTGGCGATTCAGCGTTCACTGCAG
PHR2_ E353A-Rv	CTGCAGTGAACGCTGAATCGCCAGCTGTTCATGCAGGCG
PHR2_ K292A-Fw	CTGCGCCTGCAGCTGGAACTGCAGGATCGCCTGCATGAACAGCTGGAAATTC
PHR2_ K292A-Rv	GAATTTCCAGCTGTTCATGCAGGCGATCCTGCAGTTCCAGCTGCAGGCGCAG
PHR2_ K292D-Fw	CTGACCATTTATCATGTGGATAGTCATCTGCAGAAATATCGCAC
PHR2_ K292D-Rv	GTGCGATATTTCTGCAGATGACTATCCACATGATAAATGGTCAG
PHR2_ S293A-Fw	CTGACCATTTATCATGTGAAAGCCCATCTGCAGAAATATCGCACCGCC
PHR2_ S293A-Rv	GGCGGTGCGATATTTCTGCAGATGGGCTTTCACATGATAAATGGTCAG
PHR2_ Q296A-Fw	CATGTGAAAAGTCATCTGGCCAAATATCGCACCGCCCGTTATCG
PHR2_ Q296A-Rv	CGATAACGGGCGGTGCGATATTTGGCCAGATGACTTTTCACATG
**PCR products were cloned into pBB75 vector**	
$SPX2_{1\text{-}280}$-Fw	GAAGGAGATATACATATGAAATTTGGCAAAAGCCTGTCATC
$SPX2_{1\text{-}280}$-Rv	ATCTAGAGCTCGAATTCTTA ACGACCCGGTTCATCCTGACC
SPX2 $_{1\text{-}202}$-Fw	GAAGGAGATATACATATGAAATTTGGCAAAAGCCTGTCATC
SPX2 $_{1\text{-}202}$- Rv	ATCTAGAGCTCGAATTCTTAACCATCTTCGCTTGCCACGCTATG
$SPX2_{1\text{-}267}$-Fw	GAAGGAGATATACATATGAAATTTGGCAAAAGCCTGTCATC
$SPX2_{1\text{-}267}$-Rv	ATCTAGAGCTCGAATTCTTA CGGCAGGCTAAACACGCTCAC
SPX2_ Δ47-59-Fw	GCGGCAGCCGCAGGTGGCATGACCCTG
SPX2_ Δ47-59-Rv	TTTGCTGGCACGTTCACCGGCCGC
SPX2_ Δ47-64-Fw	GGCATGACCCTGGAACAGGCAGGTTTTGTG
SPX2_ Δ47-64-Rv	TTTGCTGGCACGTTCACCGGCCGCGCCACC
$mH2A1.1_{181\text{-}366}$-Fw	CATAGCGTGGCAAGCGAAGATGGT
$mH2A1.1_{181\text{-}366}$-Rv	ATCTAGAGCTCGAATTCTTACAGTTTGGCCATTTCCTGCAC

SPX2_ Y25F-Fw	GATAATTTTCTGAGTTTTAAAGATCTGAAAAAACGTC
SPX2_ Y25F- Rv	GATAATTTTCTGAGTTTTAAAGATCTGAAAAAACGTC
SPX2_ Y25A- Fw	GATAATTTTCTGAGTGCGAAAGATCTGAAAAAACGTC
SPX2_ Y25A-Rv	GACGTTTTTTCAGATCTTTCGCACTCAGAAAATTATC
SPX2_ L28A-Fw	CTGAGTTATAAAGATGCGAAAAAACGTCTGAATCTG
SPX2_ L28A- Rv	CAGATTCAGACGTTTTTTCGCATCTTTATAACTCAG
SPX2_ K29A- Fw	AGTTATAAAGATCTGGCGAAACGTCTGAATCTG
SPX2_ K29A-Rv	CAGATTCAGACGTTTCGCCAGATCTTTATAACT
SPX2_ K143A/K147A-Fw	ACCGGTCTGGTGGCGATTCTGAAAGCGTATGATAAACGT ACC
SPX2_ K143A/K147A-Rv	GGTACGTTTATCATACGCTTTCAGAATCGCCACCAGACC GGT
SPX2_ K26A-Fw	CTGAGTTATGCTGATCTGAAAAAACGTCTG
SPX2_ K26A -Rv	CAGACGTTTTTTCAGATCAGCATAACTCAG
SPX2_ R31A-Fw	GATCTGAAAAAAGCTCTGAATCTGATTAGCGG
SPX2_ R31A -Rv	CCGCTAATCAGATTCAGAGCTTTTTTCAGATC
SPX2_ K143A-Fw	ACCGGTCTGGTGGCGATTCTGAAAAAATATG
SPX2_ K143A-Rv	CATATTTTTTCAGAATCGCCACCAGACCGGT
SPX2_ K146A-Fw	CTGGTGAAAATTCTGGCGAAATATGATAAACGTACC
SPX2_ K146A-Rv	GGTACGTTTATCATATTTCGCCAGAATTTTCACCAG
SPX2_ K147A -Fw	GTGAAAATTCTGAAAGCGTATGATAAACGTACC
SPX2_ K147A -Rv	GGTACGTTTATCATACGCTTTCAGAATTTTCAC
SPX2_ K150A-Fw	CTGAAAAAATATGATGCGCGTACCGGTAGCATG
SPX2_ K150A-Rv	CATGCTACCGGTACGCGCATCATATTTTTTCAG
SPX2_ W18A-Fw	GTGGAAATGCAGCCGGAAGCGCGCGATAATTTTCTGAG
SPX2_ W18A-Rv	CTCAGAAAATTATCGCGCGCTTCCGGCTGCATTTCCAC
SPX2_ F84A-Fw	GCGGAACTGGATAAAGCGAATTTTTTTTTTCTGG
SPX2_ F84A-Rv	CCAGAAAAAAAAAATTCGCTTTATCCAGTTCCGC
SPX2_ F87A-Fw	GCGGAACTGGATAAATTTAATTTTGCCTTTCTGG
SPX2_ F87A-Rv	CCAGAAAGGCAAAATTAAATTTATCCAGTTCCGC
SPX2_ Y133A-Fw	GTGCTGCTGGAAAATGCTAGCGCACTGAATTATACC
SPX2_ Y133A-Rv	GGTATAATTCAGTGCGCTAGCATTTTCCAGCAGCAC
SPX2_ Y133F-Fw	GTGCTGCTGGAAAATTTTAGCGCACTGAATTATACC
SPX2_ Y133F-Rv	GGTATAATTCAGTGCGCTAAAATTTTCCAGCAGCAC
SPX2_ N137A-Fw	TAGCGCACTGGCCTATACCGGTCTGGTG
SPX2_ N137A-Rv	CACCAGACCGGTATAGGCCAGTGCGCTA
SPX2_ R105E-Fw	GAACTGCGTGAAGAAAAAATGGCAAGCGCAG
SPX2_ R105E-Rv	CTGCGCTTGCCATTTTTTCTTCACGCAGTTC
SPX2_ E112R-Fw	TGGCAAGCGCAGAACGTGTGATGCGTGTG
SPX2_ E112R-Rv	CACACGCATCACACGTTCTGCGCTTGCCA
SPX2_ E119R-Fw	GTGATGCGTGTGCGTAAACGTATTGTGGATCTG
SPX2_ E119R-Rv	CAGATCCACAATACGTTTACGCACACGCATCAC
SPX2_ R19A-Fw	ATGCAGCCGGAATGGGCTGATAATTTTCTGAG
SPX2_ R19A-Rv	CTCAGAAAATTATCAGCCCATTCCGGCTGCAT

SPX2_ E93K-Fw	CTGGAAAAAGAAAAAGAATATGTGATTAAACAG
SPX2_ E93K-Rv	CTGTTTAATCACATATTCTTTTTCTTTTTCCAG
SPX2_ K100E/E101K/E104K-Fw	GTGATTAAACAGGAAAAACTGCGTAAACGTAAAATGGCAA
SPX2_ K100E/E101K/E104K-Rv	TTGCCATTTTACGTTTACGCAGTTTTTCCTGTTTAATCAC
SPX2_ K100A/E101A/E104A-Fw	AAACAGGCCGCCCTGCGTGCCCGTAAAATGGCAA
SPX2_ K100A/E101A/E104A-Rv	TTGCCATTTTACGGGCACGCAGGGCGGCCTGTTT
SPX2_ H124A-Fw	GGATCTGGCAGGTGAAATGGTGCTGC
SPX2_ H124A-Rv	GCAGCACCATTTCACCTGCCAGATCC
SPX2_ E183K-Fw	CTGGTGAAAGAATGCAAAGAAATGCTGGATCAG
SPX2_ E183K-Rv	CTGATCCAGCATTTCTTTGCATTCTTTCACCAG
SPX2_ D187K-Fw	ATGCGAAGAAATGCTGAAACAGCTGATGCCGACCAA
SPX2_ D187K-Rv	TTGGTCGGCATCAGCTGTTTCAGCATTTCTTCGCAT
SPX2_ E131R-Fw	GGTGAAATGGTGCTGCTGCGTAATTATAGCGCACTG
SPX2_ E131R-Rv	CAGTGCGCTATAATTACGCAGCAGCACCATTTCACC
SPX2_ K106C-Fw	GATTAAACAGAAAGAACTGCGTGAACGTTGCATGGCAAGCGCAGAAGAAGTGATGCG
SPX2_ K106C-Rv	CGCATCACTTCTTCTGCGCTTGCCATGCAACGTTCACGCAGTTCTTTCTGTTTAATC
SPX2_ C182S-Fw	AAACTGGTGAAAGAAAGCGAAGAAATGCTGGAT
SPX2_ C182S-Rv	ATCCAGCATTTCTTCGCTTTCTTTCACCAGTTT
**PCR products were cloned into pET15D vector**	
AtSPX2$_{1\text{-}287}$-Fw	AAGTTGATGCACATATGAAGTTCGGTAAAAGCCTGAG
AtSPX2$_{1\text{-}287}$-Rv	CAGCCGGATCCTCGAGTTATTTGGCAACCTGTTCCAGCAC
AtSPX4$_{1\text{-}318}$-Fw	AAGTTGATGCACATATGAAGTTCGGTAAAGAATTTCG
AtSPX4$_{1\text{-}318}$-Rv	CAGCCGGATCCTCGAGTTAATGACTCGGACCGGTATCTTC
SPX1$_{1\text{-}295}$-Fw	AAGTTGATGCACATATGAAATTTGGCAAAAGCCTGAG
SPX1$_{1\text{-}295}$-Rv	CAGCCGGATCCTCGAGTTATTTTGCAGCCTGTTCAATCAC
SPX4$_{1\text{-}320}$-Fw	AAGTTGATGCACATATGAAATTTGGCAAAGATTTTCGC
SPX4$_{1\text{-}320}$-Rv	CAGCCGGATCCTCGAGTTATTCATCGCGCGGCTGACCTTC
SPX4_ E87R-Fw	GATTTTTATATTGAACGTGAACGCTGGTATGTGATTCGTCTGC
SPX4_ E87R -Rv	GCAGACGAATCACATACCAGCGTTCACGTTCAATATAAAAATC
SPX4_ Q94A/V95A/E98A-Fw	GGTATGTGATTCGTCTGGCGGCGCTGAAAGCGCGCATTGAACGTGTTAA
SPX4_	TTAACACGTTCAATGCGCGCTTTCAGCGCCGCCAGACG

Q94A/V95A/E98A-Rv	AATCACATACC
SPX4_ H132A-Fw	CGCAAAGCCTTTGTTATTATTGCGGGCGAAATGATTCTGCTGC
SPX4_ H132A-Rv	GCAGCAGAATCATTTCGCCCGCAATAATAACAAAGGCTTTGCG
SPX4_ E191K-Fw	CGCCTGGTGCGTGAATGTAAAGCGAATCTGGAACTGCTG
SPX4_ E191K-Rv	CAGCAGTTCCAGATTCGCTTTACATTCACGCACCAGGCG
SPX4_ E195K-Fw	GAATGTGAAGCGAATCTGAAACTGCTGTTTCCGATTGAAGC
SPX4_ E195K-Rv	GCTTCAATCGGAAACAGCAGTTTCAGATTCGCTTCACATTC
SPX4_ Q139R-Fw	CATGGCGAAATGATTCTGCTGCGCACCTATTCTAGTCTGAATTTT
SPX4_ Q139R-Rv	AAAATTCAGACTAGAATAGGTGCGCAGCAGAATCATTTCGCCATG

Fw and Rv represent a forward primer and a reverse primer, respectively.

References

1 Desmarini, D., Lev, S., Furkert, D., Crossett, B., Saiardi, A., Kaufman-Francis, K., Li, C., Sorrell, T. C., Wilkinson-White, L., Matthews, J., Fiedler, D. & Djordjevic, J. T. IP7-SPX Domain Interaction Controls Fungal Virulence by Stabilizing Phosphate Signaling Machinery. *mBio* **11** (2020).

2 Corpet, F. Multiple sequence alignment with hierarchical clustering. *Nucleic Acids Res* **16**, 10881-10890 (1988).

3 Jiang, M., Sun, L., Isupov, M. N., Littlechild, J. A., Wu, X., Wang, Q., Wang, Q., Yang, W. & Wu, Y. Structural basis for the Target DNA recognition and binding by the MYB domain of phosphate starvation response 1. *FEBS J* **286**, 2809-2821 (2019).

4 Ried, M. K., Wild, R., Zhu, J., Pipercevic, J., Sturm, K., Broger, L., Harmel, R. K., Abriata, L. A., Hothorn, L. A., Fiedler, D., Hiller, S. & Hothorn, M. Inositol pyrophosphates promote the interaction of SPX domains with the coiled-coil motif of PHR transcription factors to regulate plant phosphate homeostasis. *Nat Commun* **12**, 384 (2021).
